# Comparison of Whole-Cell SELEX Methods for the Identification of *Staphylococcus Aureus*-Specific DNA Aptamers

**DOI:** 10.3390/s150408884

**Published:** 2015-04-15

**Authors:** Jihea Moon, Giyoung Kim, Saet Byeol Park, Jongguk Lim, Changyeun Mo

**Affiliations:** National Academy of Agricultural Science, 310 Nongsaengmyeng-ro, Wansan-gu, Jeonju 560500, Korea; E-Mails: mmir95@gmail.com (J.M.); veryvenus0911@gmail.com (S.B.P.); limjg@korea.kr (J.L.); cymoh100@korea.kr (C.M.)

**Keywords:** aptamer, biosensor, pathogen detection, whole-cell SELEX

## Abstract

Whole-cell Systemic Evolution of Ligands by Exponential enrichment (SELEX) is the process by which aptamers specific to target cells are developed. Aptamers selected by whole-cell SELEX have high affinity and specificity for bacterial surface molecules and live bacterial targets. To identify DNA aptamers specific to *Staphylococcus aureus*, we applied our rapid whole-cell SELEX method to a single-stranded ssDNA library. To improve the specificity and selectivity of the aptamers, we designed, selected, and developed two categories of aptamers that were selected by two kinds of whole-cell SELEX, by mixing and combining FACS analysis and a counter-SELEX process. Using this approach, we have developed a biosensor system that employs a high affinity aptamer for detection of target bacteria. FAM-labeled aptamer sequences with high binding to *S. aureus*, as determined by fluorescence spectroscopic analysis, were identified, and aptamer A14, selected by the basic whole-cell SELEX using a once-off FACS analysis, and which had a high binding affinity and specificity, was chosen. The binding assay was evaluated using FACS analysis. Our study demonstrated the development of a set of whole-cell SELEX derived aptamers specific to *S. aureus*; this approach can be used in the identification of other bacteria.

## 1. Introduction

Enterotoxin-producing *Staphylococcus aureus* (*S. aureus*) cause nausea, stomach cramps, vomiting, and diarrhea [1]. In some cases, such as in infants, elderly or immunocompromised individuals, *S. aureus* infections can be severe [2]. *S. aureus* outbreaks have been associated with meat, poultry, and their products; however, *S. aureus* is also present in the air, in dust, water, food, humans, and animals [3]. In fact, *S. aureus* is one of the most common and important foodborne bacteria in the world [4]. In Korea, *S. aureus* caused 6.8% of foodborne illnesses recorded during 2002–2012 [5]. In the USA, 241,188 illnesses, 1064 hospitalizations, and six deaths due to staphylococcal food poisoning are reported annually [6].

Recently, the control of *S. aureus* outbreaks has gained social and economic importance globally [3] emphasizing the need for a rapid method of detecting *S. aureus*. Although methods for detection and control of *S. aureus* are well established, conventional methods, such as the standard plate count method after selective enrichment in broth, are lengthy, complicated and require multiple steps [4,7]. To overcome these factors, biosensors that allow rapid and efficient detection of foodborne bacteria have been developed [8,9]. Biosensors are composed of a bioreceptor, transducer, and a data recording and display device. In this context, a bioreceptor is a biological molecular species, such as an antibody, enzyme, protein, or nucleic acid that utilizes a biochemical mechanism for the recognition of a biological molecule [10]. Antibodies have commonly been used as bioreceptors that exhibit high affinity and a specific binding capability between antigen and antibody. However, the use of antibodies has notable weaknesses, such as requiring animal hosts, although they are not well tolerated, as well as a very expensive and laborious production process, batch-to-batch variation, sensitivity to temperature, irreversible denaturation, and limited shelf life [11].

Consequently, aptamers have been developed that can be used instead of antibodies. Aptamers are composed of ssDNA or RNA, and exhibit highly selective and specific binding affinities for target molecules [12]. Aptamers, shorter than 40 nucleotides, are produced with great accuracy and reproducibility, by chemical synthesis, and are stable across temperatures and during long-term storage [13]. Aptamers are identified by means of Systematic Evolution of Ligands by EXponetial enrichment (SELEX). SELEX is a technique by which random libraries of oligonucleotides can be screened by *in vitro* selection using targets and can then be amplified by PCR [12]. Among SELEX technologies, whole-cell SELEX is a method developed and modified for creating aptamers bound to live bacteria [14]. Whole-cell SELEX comprises multiple steps: (1) screening of random nucleic acid bound to target bacteria, (2) repeated separation and exponential amplification of the oligonucleotide, and (3) cloning and sequencing of the specific binding molecules ultimately identified [15]. Because the target in whole-cell SELEX is live pathogenic bacteria, aptamers can find and bind more efficiently to the surface molecules of live bacteria than those in other SELEX approaches [16]. Therefore, the aptamers derived in whole-cell SELEX may have little cross-reactivity to non-target bacteria [17].

Despite of the advantages of whole-cell SELEX, its success rate is only below 50% as to the complex structure of target bacteria [18]. Therefore, to improve development of successful aptamers, various SELEX processes have emerged, designed to suit the requirements of specific individual purposes [19]. In this study, we designed, developed, and characterized DNA aptamers specific to *S. aureus*, using two kinds of modified whole-cell SELEX approaches for improving the specificity and selectivity of the aptamers. Because flow cytometry is a crucial method for selecting target aptamers specifically bound to cells [20], multiple FACS sorting steps were incorporated in the modified SELEX process to further improve the specificity. We then compared their binding activity using FACS analysis.

## 2. Materials and Methods 

### 2.1. Biological and Chemical Materials

*Salmonella typhimurium* (KCCM 12041), *Salmonella* *enteritidis* (KCCM 12021), *Escherichia* *coli* (KCCM 11234), and *Staphylococcus* *aureus* (KCCM 12103) were obtained from the Korean Collection of Type Cultures (Daejun, Korea). Tryptic soy (TS) agar, BBL eosin methylene blue (EMB) agar, XLT4 agar base, XLT4 agar supplement, Baird–Parker agar base, and EY tellurite enrichment were purchased from BD Difco (Sparks, MD, USA). The initial ssDNA library and the primers used for amplification were synthesized and purified by polyacrylamide gel electrophoresis (PAGE; Bioneer Co., Ltd, Daejeon, Korea). Phosphate–buffered saline (PBS, pH 7.4) was purchased from Sigma (St. Louis, MO, USA). PCR tubes, reagents, and polymerase were obtained from Takara (Shiga, Japan). LE agarose and TAE buffer were purchased from Lonza (Rockland, ME, USA). The Qiagen MinElute gel extraction kit was obtained from Qiagen (Hilden, Germany). The In-Fusion HD Cloning kit was purchased from Clontech (Mountain View, CA, USA). 

### 2.2. Bacterial Strains

Stock cultures of *S. aureus*, *S*. *typhimurium*, *S*. *enteritidis*, and *E. coli* were grown overnight at 35 °C in brain–heart infusion medium (Difco, Franklin Lakes, NJ, USA). Cells were harvested by centrifugation, washed three times in PBS, and finally suspended in 100 µL of PBS prior to use in experiments.

### 2.3. Preparation of DNA Library

The DNA template was synthesized as ssDNA containing 40 random nucleotides, viz. 5′-CGGATGCGAATTCCCTAATACGACTCACTATAGGGCGT–N_40_–GGTGGATCCATATTCCTACTCG-3′. The initial DNA library was prepared by PCR amplification with Pyrobest DNA polymerase (Takara).

### 2.4. PCR Optimization 

PCR was performed prior to binding of the SELEX library to *S. aureus* to optimize the annealing temperature. One hundred µL PCR mix consisting of 1 ng of either capture of reporter SELEX DNA templates, 25 pmol of appropriate primer (unlabeled or FAM[fluorescein]–labeled 20mer primers), 10 µL of 10× Pyrobest buffer, 10 mM dNTP (Takara), 2 units of Pyrobest DNA polymerase, and double distilled water (ddH_2_O; Biosesang, Gyeonggi-do, Korea) was used for each PCR in a thermal cycler (BioRad, Hercules, CA, USA). PCR annealing temperature was studied between 56 °C and 70 °C (data not shown). Optimal PCR conditions were determined to be an initial denaturation at 98 °C for 2 min, followed by 30 cycles, each consisting of 98 °C for 30 s, 67 °C for 15 s, 72 °C for 30 s, and a final extension at 72 °C for 5 min.

### 2.5. SELEX Procedures 

**Figure 1:**
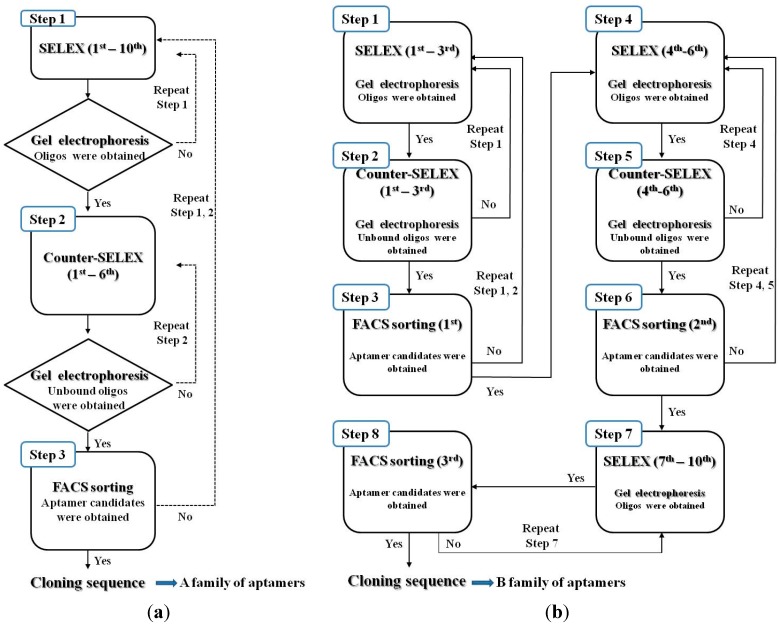
(**a**) Schematic diagram of the basic whole-cell SELEX procedures used to isolate DNA aptamers with high binding affinity for *Staphylococcus aureus*; (**b**) Schematic diagram of the modified whole-cell SELEX process used to isolate DNA aptamers with a high binding affinity for *Staphylococcus aureus*.

The whole-cell SELEX process is outlined in Figure 1. To improve the selectivity of the aptamer, SELEX was designed using two types of processes. A total of 10 rounds of SELEX and 6 rounds of counter-SELEX were performed. In case of Figure 1a, the sorting step using flow cytometry, was conducted after all the SELEX stages had been completed. In the case described in Figure 1b, the aptamer candidates for binding to *S. aureus* were sorted using flow cytometry after the third, sixth, and tenth rounds of SELEX or counter-SELEX. SELEX was performed using a modification of Moon’s methods [21]. 

In the selection rounds, 1.0 µg of double-stranded DNA library, dissolved in 50 µL of PBS (pH 7.4, Sigma, St. Louis, MO, USA) was denatured by heating at 95 °C for 10 min and then cooling on ice for 10 min. The denatured ssDNA was incubated with 100 µL of 1.0 × 10^8^ cfu/mL *S. aureus*, with shaking (1500 rpm) at room temperature for 45 min. Unbound ssDNA was removed by centrifugation at 8000 g for 10 min. Then, the bound ssDNA was eluted in 500 µL of double distilled water (ddH_2_O) by heating bacteria–DNA complexes at 95 °C for 10 min. After centrifugation, the supernatant was used as the aptamer candidate template for amplification by PCR. Electrophoresis on a 2.0% agarose gel was used to confirm the purity and to verify the size of the PCR products. Some of the PCR products, insufficiently amplified to allow detection of the band size on the Gel-doc system (BioRad), were purified by Ultracel (Milipore, Billerca, MA, USA). After verification of the band on a gel, the target materials were carefully excised and extracted using a MinElute gel extraction kit (Qiagen, Hilden, Germany). These extraction methods were also used in the rounds of counter-selection. After incubation with non-target bacteria, bound ssDNA was removed by centrifugation, while the unbound aptamer candidates in the supernatant were collected for one more round of counter-SELEX or SELEX. In case of PCR failure caused by human errors or unknown reasons, aptamer candidates selected from the previous step were used for the remaining process.

### 2.6. Fluorescence-Activated Cell Sorting (FACS)

Cell sorting was performed after the third, sixth, and tenth rounds of counter-SELEX or SELEX. For cell sorting, the selected DNA was fluorescently labeled via PCR amplification with 5'-FAM- and 3'-FAM-modified primers. The FAM-dsDNA was denatured to ssDNA by heat treatment. Binding assays involved incubating 1.0 µg of FAM-labeled ssDNA with 10^8^ cells of *S. aureus* in PBS for 45 min and then washing twice with PBS. Cells were resuspended in 100 µL of PBS for flow cytometric analysis. Forward scatter, side scatter, and fluorescence intensity were measured and gated fluorescence intensity above background level was quantified. Aptamer candidates bound to *S. aureus* were sorted by FACS and then amplified with non-labeled primers. These amplified aptamer candidates were cloned and sequenced at Macrogen (Seoul, Korea). The aptamer sequences were analyzed using a commercial genetic analysis program (CLC Workbench 6, Aarhus, Denmark).

### 2.7. Characterization of Binding Parameters of Aptamers to Bacteria 

To test the binding affinity of aptamer candidates, fluorescence analysis was performed using an Infinite M1000 spectrophotometer (TECAN, Männedorf, Switzerland). The binding affinity of aptamer candidates was determined by incubating *S. aureus* cells (10^8^) with varying concentrations of FAM-labeled aptamer candidates in 500 µL of PBS for 45 min at room temperature. Cells were then washed twice with 500 µL of PBS, suspended in 100 µL of PBS, and then analyzed. The FAM-labeled unselected ssDNA library was used as a negative control for non-specific binding. All the binding assay experiments were repeated 3 times. The mean fluorescence intensity of target bacteria labeled with aptamer candidates was used to calculate the specific binding by subtracting the mean fluorescence intensity of nonspecific binding produced by unselected library DNA. The equilibrium dissociation constants (*K_d_*) of the fluorescent ligands were obtained by fitting the dependence of the fluorescence intensity of specific binding on the concentration of the ligands to the equation *Y = B_max_X* / (*K_d_ + X*) using Graphpad Prism 6 (GraphPad Software, CA, USA).

### 2.8. Aptamer-Binding Assays and Prediction of Candidate Aptamer Sequences 

Aptamer A14 was evaluated for binding activity and cross reactivity with related and unrelated pathogenic bacteria. The FAM-labeled aptamer (1.0 nmol) was dissolved in 50 µL of ddH_2_O, and denatured by heating (95 °C for 10 min) and cooling (on ice for 10 min). Then, 1.0 nmol of FAM-labeled aptamer was incubated with target bacteria (10^8^ CFU) in 500 µL of PBS for 45 min at room temperature. After incubation, samples were centrifuged at 8000× g for 10 min and the supernatant removed. Five hundred microliters of PBS was added to the samples, which were then mixed well. After one more PBS wash step, 50 µL of PBS was added for the binding assay. The fluorescently labeled analytes were monitored at 505–535 nm, using an Infinite M1000 microplate reader (TECAN) at an excitation of 494 nm. To evaluate cross-reactivity, the binding assays were repeated using *E. coli*, *S. typhimurium*, and *S. enteritidis*. For further characterization of binding affinity, different concentrations of FAM-aptamers (0.1, 0.3, 0.5, 0.7, and 3 nmol) were used and analyzed by FACS. FACS analysis was performed on 20,000 cells. The secondary structure of aptamers binding to *S. aureus* was predicted using the genetic analysis program (CLC Workbench 6).

## 3. Results and Discussion

### 3.1. Screening of Aptamer Candidates in the Basic Whole-Cell SELEX

**Figure 2:**
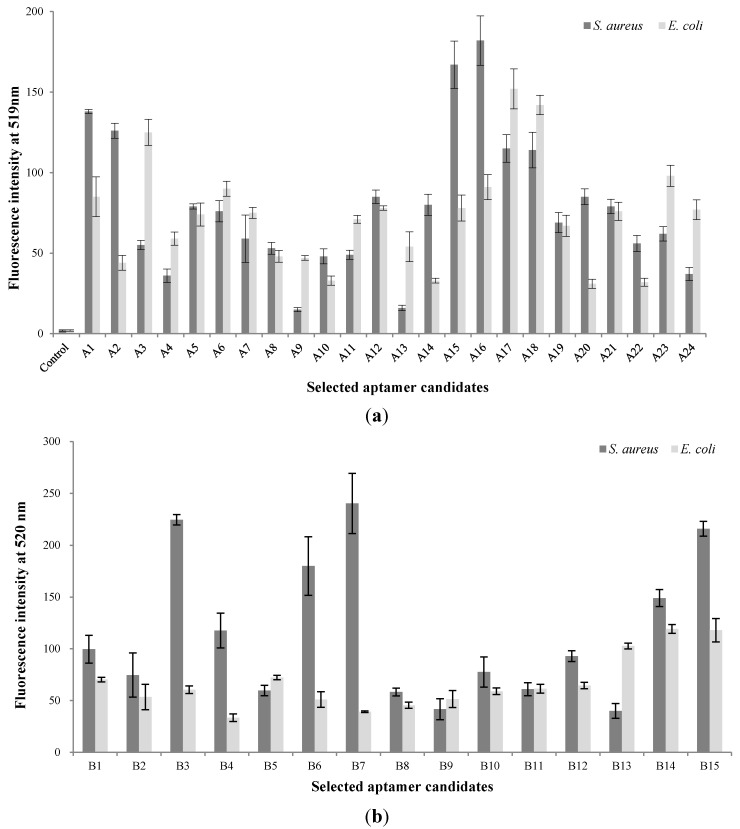
(**a**) Validation of the binding specificity of selected aptamer candidates by using the basic whole-cell SELEX procedure; (**b**) Validation of binding specificity of selected aptamer candidates by using the modified whole-cell SELEX procedure.

A total of 10 rounds of SELEX and 6 rounds of counter-SELEX were performed to select aptamers with selectivity for *S. aureus*. Cell-bound aptamers were separated by cell sorting using FACS. The selected aptamer candidates were cloned and sequenced (Figure 1a). From these, the majority of sequences could be classified into 24 families. These unique aptamer candidates were pre-screened for binding interaction with *S. aureus* and *E. coli* cells (10^7^) by using 1.0 nmol of each sequence by fluorescence spectrometry (Figure 2a). A15 showed the highest fluorescence, followed by A14. Although comparatively lower affinity for *S. aureus* was shown by aptamer A2 and A20, they also showed lower affinity to *E. coli*. The sequences selected from the randomized region of A2, A14, A15 and A20 are in Table 1. Error bars on each graph indicate the standard deviation of fluorescence signals from three samples. 

**Table 1:** Sequences of selected aptamers for *Staphylococcus aureus*.

Aptamers	Sequences
A2	ACGGGCGTGGGAGGCAATGCCTTGCTTGTAGGCTTCCCCTGTGCGCG
A14	CACACCGCAGCAGTGGGAACGTTTCAGCCATGCAAGCATCACGCCCGT
A15	CACGCGCAAACAGATTAACACTCCGCCTAAGTCTGCCGCACGC
A20	GCGTGCAGCGGGGGCTGCGCGGTGGAGTGCTGTGGGCG
B3	GCGTGCGGAGCCAGGATGGGAGGTCTGTAGGTCTGCGGGGCGTG
B6	GCGTGTCGGTGTCTGCCGGGGGATGTGGAGGCTGGGTGTTGCGCG
B7	GCGTGGGCGGGCTACCTGGCTAGTACGCCATGATGCCTGCACGCG
B15	CACGCGCAAACAGATTAACACTCCGCCTAAGTCTGCCGCACGC

FACS analysis is an important method for developing aptamers specifically bound to target cells [22]. According to a previous study, nucleic acids (aptamer candidates), selected by whole-cell SELEX, would have good affinity for, but also poor specificity to target bacteria. Thus, FACS analysis could be a crucial step in improving their affinity and specificity. We compared the selective intensity and specificity of aptamers A2, A14, A15, and A20 (1.0 nmol each) for *S. aureus* and *E. coli* (10^8^ cells each) by flow cytometry. The control groups were *S. aureus*, and *E. coli* to which no ssDNA had been added. Compared with the *S. aureus* control group, the selected aptamer candidates demonstrated good binding affinity. The selected aptamers A14 and A15, demonstrated stronger binding affinity for *S. aureus* than did A2 and A20 (Figure S1). However, A15 showed some binding affinity for *E. coli* as compared with the *E. coli* control group.

### 3.2. Screening of Aptamer Candidates in the Modified Whole-Cell SELEX

In the case of the modified whole-cell SELEX, the SELEX process was repeated with SELEX *per se*, cell sorting by FACS analysis, and counter-SELEX for improving the specificity and selectivity of aptamer. Our SELEX approach is summarized in Figure 1b. First, three rounds of SELEX and counter-SELEX were conducted for screening the aptamer candidates, specific to target bacteria. The first cell sorting was performed as described and sorted DNAs were amplified for the next step of the process [21]. Second, three rounds of counter-SELEX and SELEX were carried out after each other. After six rounds of SELEX, the second cell sorting step was performed. Third, three rounds of counter-SELEX and four rounds of SELEX were performed, and then the last cell sorting step was used for retaining the aptamer candidates. Using *S. aureus* as target cell and *S*. *typhimurium* and *E. coli* as negative control for counter-selection, we generated aptamers targeting molecules only present on the cell surface of *S. aureus* [16]. In addition, we added multiple cell sorting steps to obtain aptamers that are reliable in whole-cell SELEX. Therefore, our modified whole-cell SELEX process combined with cell sorting and counter-SELEX allowed us to optimize selectivity and specificity over the established SELEX method.

Moreover, in this study, the entire DNA library not bound to *S. aureus* was removed in the repeated selection and cell-sorting process. Observation of a target band on the agarose gel after the rounds of selection suggested that aptamer candidates could specifically bind to *S. aureus*. After the selection process, the sorted aptamer pools were cloned and sequenced, resulting in a total of 34 sequences. According to the sequencing results and the homology of the DNA sequences, the majority of the sequences could be classified into 15 families. These aptamer candidates were synthesized and modified with FAM, to allow determination of the binding affinity by fluorescent spectrophotometry. Spectrophotomeric analysis has recently been used to enhance the ease, rapidity, and sensitivity of the process [20,21,23]. The selected FAM-dsDNA aptamer candidates were denatured by heat treatment for transferring to FAM-ssDNA. Their sequences selected from the randomized region are shown in Table 1. They were bound to *S. aureus*, and were then analyzed and compared. To characterize the specificity of aptamer candidates, FAM-labeled aptamers were tested against *E. coli*. Aptamer B7 showed the highest florescence, followed by B3, B6, and B15, while comparatively lower affinity was shown by aptamer B9 and B13 (Figure 2b).

Aptamer B3, B6, B7, and B15 were analyzed by FACS using the same methods as those in basic SELEX. Aptamer B3 showed the highest binding affinity for *S. aureus*, followed by B15, B6, and B7. Aptamer B7 demonstrated a low specificity for *E. coli* (non-target bacteria), but the remaining aptamers showed a similar low detection level for this organism (Figure S2). According to the binding affinity and selectivity as determined by FACS analysis, aptamer A14, B3, and B6 were selected as showing specific binding to *S. aureus*. Although the exclusivity of selected aptamers was low with common Gram-negative foodborne pathogens, there might be higher cross reactivity with Gram-positive bacteria because of the similarities in cell structure.

When we compared the selective affinity of aptamers between the basic and modified SELEX procedures, the binding affinities of group A were superior to those of group B, but their selectivity was less. Although the SELEX combined with FACS analysis would be expected higher binding affinity to more than that obtained with basic SELEX, it was not agreed selectivity and specificity of aptamers in this study. This trend is likely due to the random selectivity of the aptamer [16]. SELEX involves selection from a random DNA library [24]; in our case, the specific affinities of the DNA library could be increased by FACS, but the binding affinities would be decreased through the same process. In order to compare the efficacy between basic and modified SELEX, we then analyzed the dissociation constants of the aptamers.

### 3.3. Dissociation Constants for Aptamers 

**Figure 3:**
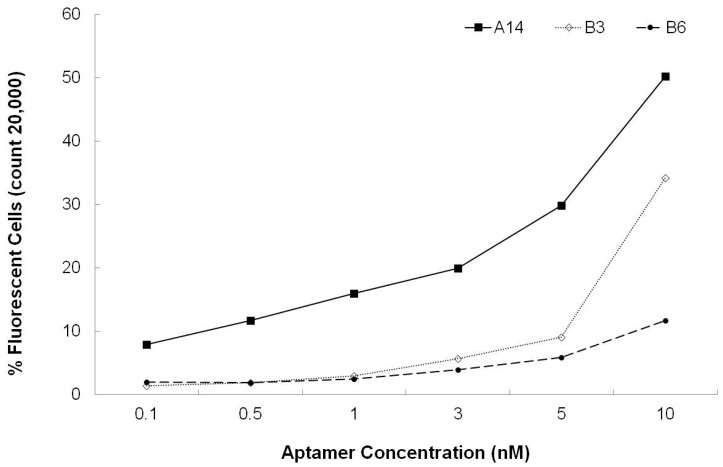
Characterization of aptamers A14, B3, and B6 binding.

Prior to selection of the leading aptamer candidate, the percent binding efficiency of the aptamer for binding to 10^8^ *S. aureus* cells was evaluated. Aptamer A14 showed the highest binding rate (50.18% cells) at an aptamer concentration of 10 nM, but other aptamers showed a lower binding rate (Figure 3). 

**Figure 4:**
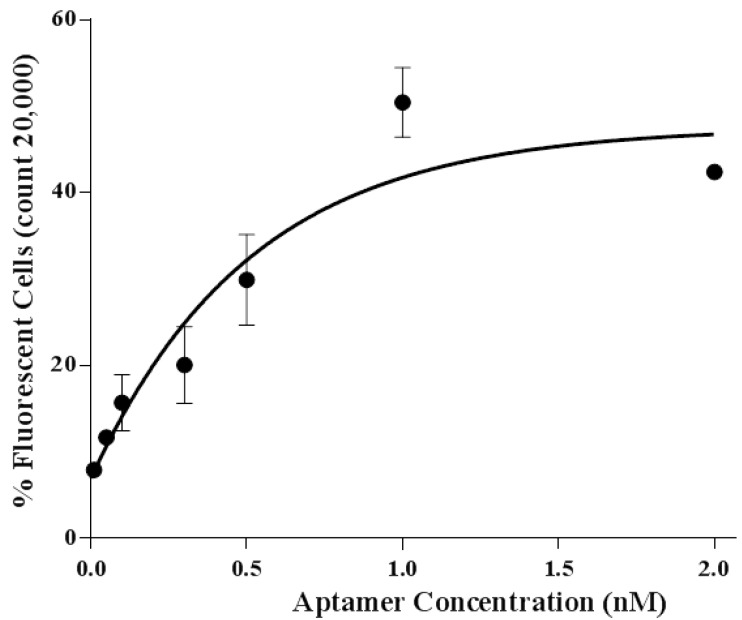
Binding affinity analysis of aptamer A14.

In this study, aptamer A14 demonstrated the highest binding affinity, and its equilibrium dissociation constant (Kd) was 3.49 ± 1.43 nM (Figure 4). These results showed that the binding affinity of aptamer A14 was similar to those of established aptamers specific to *S. aureus* (Kd of SA17-GNPs and SA61-GNPs: 3.03 nM and 9.9 nM, respectively) in another study [25]. Compare to Kd values of other developed aptamers for *Listeria* spp (Kd of LM12-6 and LM6-116: 106.4 and 74.4 nM, respectively) [26] and *Salmonella* *typhiamurium* (Kd of S8-7: 1.73 µM) [16] aptamer A14 has relatively strong binding affinity.

**Figure 5:**
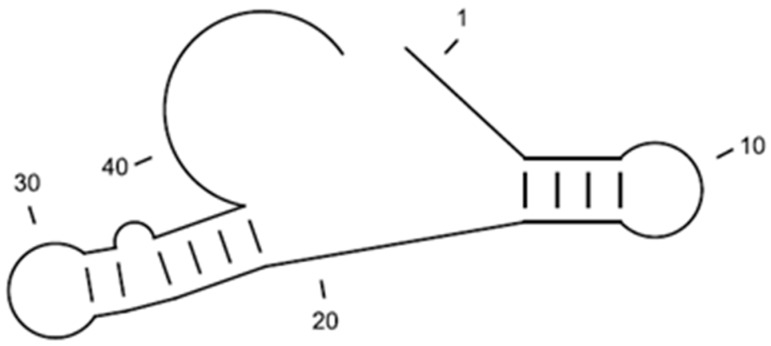
Predicted secondary structure of aptamer A14.

To further investigate aptamer A14, we analyzed its secondary structures. The predicted secondary structure indicates that aptamer A14 could form 2 stem-loop branches and a larger central loop (Figure 5).

In our previous whole-cell SELEX, *S*. *typhimurium* was used as target cell [21]. In *S. aureus* cell selection, we expanded the whole-cell SELEX strategy to a cell sorting process. Additionally, combination of separation of binding and cell sorting processes is more effective for improving the specificity of the aptamers than traditional SELEX approaches. The general SELEX process, combined with *in vitro* selection and amplification, is a technique that can be used for screening very large combinatorial libraries. This selection is divided by positive selection of target cells as well as negative selection. In some studies of aptamer-specific food pathogens, the total number of SELEX rounds were less than or equal to 10. These rounds were composed of SELEX and counter-SELEX, as follows: 3rd SELEX and 4th counter-SELEX against *S. aureus*, 10th SELEX and 2nd counter-SELEX against *C. jejuni* [27], 10th SELEX and 3rd counter-SELEX against *S.* *enteritidis* [28], 7th–10th SELEX and 2th–6th counter SELEX against *S*. *typhimurium* [16,21,29,30], and only the 8th SELEX against *Lactobacillus acidophilus* [22]. These selection processes were designed and performed to improve the specificity and selectivity for target bacteria. In these experiments, cell sorting was performed after the SELEX process. We compared the efficacy of binding of aptamers developed by the basic and modified SELEX process in this study. Our study is the first report that compares the aptamer-binding affinity between basic and modified whole-cell SELEX procedures, aimed at improving selectivity and specificity of DNA aptamers specific for *S. aureus*. This approach presents some hints for designing and optimizing the developing whole-cell SELEX.

Recently, aptamers specific to foodborne pathogens have been developed using whole-cell SELEX [11,14,15,19,21,22,27,28,30]. A total of 18 manuscripts related to the use of aptamers for the identification of *S. aureus* were found in PubMed upon searching the keywords “*S. aureus*” and “aptamer”. In these manuscripts, aptamers targeting *S. aureus* cells were selected and identified, by means of whole-cell SELEX with counter-SELEX [17,25]. Among these manuscripts, no one selected and identified aptamers specific for *S. aureus* cells using FACS sorting. Our study is the first report comparing two kinds of FACS sorting based SELEX approaches to improve the selectivity and specificity of DNA aptamers specific for *S. aureus*. This approach presents an efficient manner for developing aptamers using whole-cell SELEX.

According to reports on the development of aptamers specific for bacteria, whole-cell SELEX has some advantages. First, the aptamers selected by whole-cell SELEX may have affinity for live cells over inactivated cells. Second, whole-cell SELEX could improve the binding affinity of aptamers by combining it with FACS analysis [16].

## 4. Conclusions/Outlook

FACS analysis has been used to isolate DNA bound to target cells and to remove unbound DNAs in whole-cell SELEX. Some studies have suggested that FACS analysis could be implemented in the whole-cell SELEX for perfect separation of bound from unbound sequences [18,31], also which is an important part of SELEX [21]. Considering these characteristics, we suggested two kinds of whole-cell SELEX procedures (Figure 1a,b). We assumed that the modified whole-cell SELEX was superior to the basic model, because the modified model has three FACS analysis steps. However, the binding affinity of aptamers obtained using basic whole-cell SELEX was better in the case of *S. aureus*. Given this result, we suggest that the basic whole-cell SELEX may be suitable for the development of aptamers specific to *S. aureus*. However, the limitation of this study was that it was performed using a single DNA library pool. Although we used a DNA library with a diversity of 10^14^–10^16^, this is a common way to develop aptamers specific to pathogenic bacteria [16,18,19,21,32]. The hallmark of the SELEX process is random selection from a pool. Therefore, further study would be needed to perform whole-cell SELEX in larger pools. In summary, our study has suggested the suitability of basic whole-cell SELEX for the development of aptamers specific to *S. aureus*. Moreover, aptamer A14 could be a potential ligand for capturing *S. aureus*, and may be useful in development of sensitive methodology for bacterial detection. 

## Supplementary Files

Supplementary File 1
