# Supplementary material for: Comparison of Whole-Cell SELEX Methods for the Identification of Staphylococcus Aureus-Specific DNA Aptamers
Source: Sensors (Basel). 2015 Apr 15;15(4):8884–97. doi: 10.3390/s150408884 (PMC4431181; doi:10.3390/s150408884)
Supplement: Supplementary File 1 [file sensors-15-08884-s001.pdf]

OPEN ACCESS

sensors

ISSN 1424-8220

www.mdpi.com/journal/sensors

Supplementary Information

Comparison of Whole-Cell SELEX Methods for the Identification of Staphylococcus Aureus-Specific DNA Aptamers. *Sensors* 2015, *15*, 8884-8897

Jihea Moon, Giyoung Kim *, Saet Byeol Park, Jongguk Lim and Changyeun Mo

National Academy of Agricultural Science, 310 Nongsaengmyeng-ro, Wansan-gu, Jeonju 560500, Korea; E-Mails: mmir95@gmail.com (J.M.); veryvenus0911@gmail.com (S.B.P.); limjg@korea.kr (J.L.); cymoh100@korea.kr (C.M.)

* Author to whom correspondence should be addressed; E-Mail: giyoung@korea.kr; Tel.: +82-63-238-4111; Fax: +82-63-238-4105.

Aptamer candidates	Control	A2	A14	A15	A20
with *S. aureus*	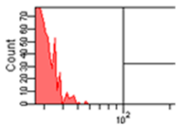	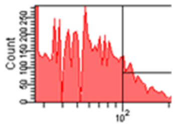	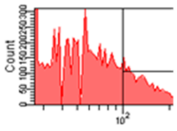	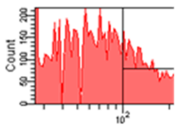	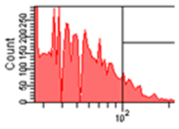
with *E. coli*	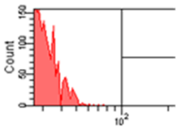	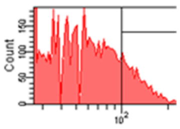	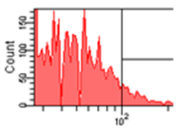	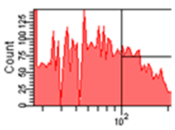	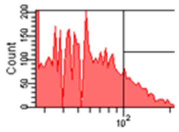

Figure S1. FACS analysis used to monitor the binding of FAM-aptamers to target/non-target bacteria in the basic whole-cell SELEX procedure.

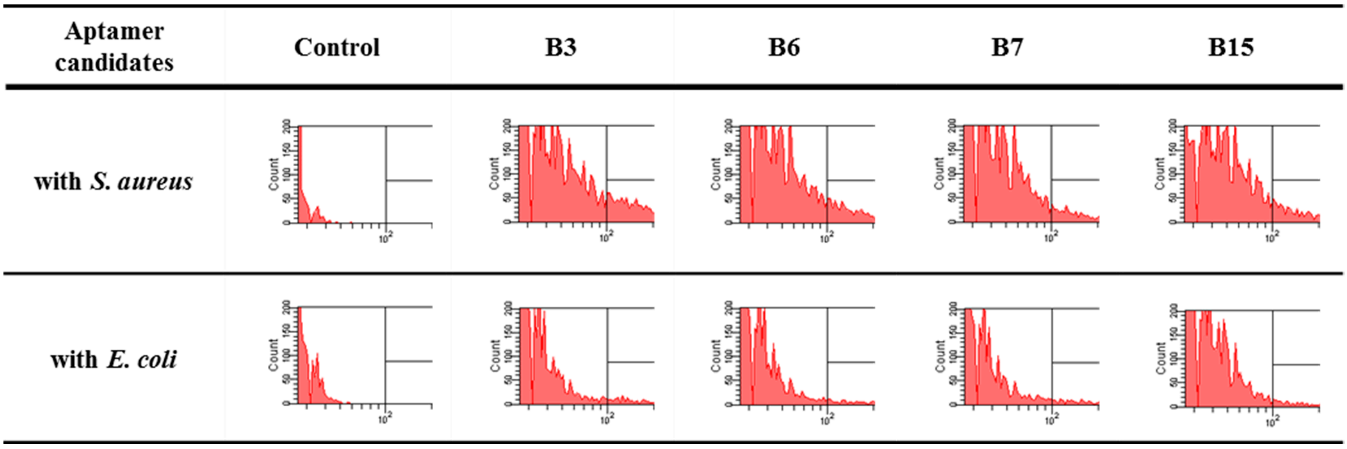

Figure S2. FACS analysis used to monitor the binding of FAM-aptamers to target/non-target bacteria in the modified whole-cell SELEX procedure.

© 2015 by the authors; licensee MDPI, Basel, Switzerland. This article is an open access article distributed under the terms and conditions of the Creative Commons Attribution license (http://creativecommons.org/licenses/by/4.0/).
